# Anesthetic Management for Encephaloduroarteriosynangiosis in Moyamoya Disease: A Hemodynamic and Neuromonitoring-Integrated Framework

**DOI:** 10.3390/jcm15134954

**Published:** 2026-06-25

**Authors:** Vikas Chauhan

**Affiliations:** Department of Anesthesiology, University of Mississippi Medical Center, 2500 North State Street, Jackson, MS 39216, USA; vchauhan@umc.edu

**Keywords:** moyamoya disease, encephaloduroarteriosynangiosis, EDAS, neuroanesthesia, cerebral perfusion, hyperventilation, normocapnia, electroencephalography, neuromonitoring, cerebral blood flow

## Abstract

Moyamoya disease is a progressive steno-occlusive cerebrovascular disorder in which cerebral perfusion may become highly dependent on systemic arterial pressure, arterial carbon dioxide tension, and collateral flow. Encephaloduroarteriosynangiosis (EDAS) is an indirect revascularization procedure that promotes neovascularization over weeks to months but does not immediately augment cerebral blood flow intraoperatively. Anesthetic management therefore requires preservation of cerebral oxygen delivery during a period of persistent physiologic vulnerability. This narrative review presents a practical perioperative framework for EDAS anesthesia, emphasizing maintenance of mean arterial pressure near baseline or modestly above baseline, avoidance of hypotension and hypovolemia, normoxia, normothermia, and careful regulation of carbon dioxide. Hyperventilation should be avoided because hypocapnia can reduce cerebral blood flow through vasoconstriction, while excessive hypercapnia may contribute to regional maldistribution or steal physiology. Raw electroencephalography may provide cortical ischemia surveillance where available, whereas somatosensory evoked potentials, motor evoked potentials, near-infrared spectroscopy, and transcranial Doppler should be considered adjunctive and institution-dependent. A structured algorithm that integrates hemodynamics, ventilation, oxygen delivery, anesthetic depth, neuromonitoring, and surgical communication may support the timely recognition and correction of intraoperative hypoperfusion.

## 1. Introduction

Moyamoya disease is a chronic, progressive steno-occlusive arteriopathy that affects the distal internal carotid arteries and the proximal anterior and middle cerebral circulation. The hallmark of the disease—the fragile basal collaterals visible on angiography as a hazy “puff of smoke”—develops as collateral channels form in response to progressive loss of major-vessel flow [1]. Patients present in widely different ways depending on age [2]. Children commonly have transient ischemic attacks, ischemic stroke, and seizures, while adults often present with intracranial hemorrhage from rupture of these collateral channels [3,4]. Cognitive change, recurrent headache, and developmental delay are also recognized presentations [5,6,7,8,9].

For symptomatic disease, surgical revascularization remains the principal disease-modifying therapy. Direct techniques such as superficial temporal artery–middle cerebral artery (STA–MCA) bypass produce early flow augmentation [10,11]. Indirect techniques, of which Encephaloduroarteriosynangiosis (EDAS)is the most familiar, depend on neovascularization of the cortex from a vascularized donor area—typically the superficial temporal artery—laid against the pia [12,13,14,15,16]. EDAS is technically less demanding than direct bypass and is widely performed in pediatric and adult Moyamoya, particularly when vessel caliber or anatomy makes direct anastomosis impractical [10,17,18]. For EDAS, the key anesthetic challenge is that the new collateral network does not provide immediate intraoperative flow augmentation. Thus, intraoperative cortical perfusion remains dependent on the patient’s pre-existing collateral circulation. Surgical benefit accrues over weeks to months [19].

That delay also outlines the anesthesia plan for EDAS. The anesthetic objective, therefore, extends beyond unconsciousness, immobility, and favorable surgical conditions; it is to preserve perfusion of an already vulnerable cerebral circulation throughout induction, scalp dissection, donor vessel mobilization, dural opening, and emergence, when autoregulatory reserve may be limited or exhausted. Brief hypotension, a sudden reduction in partial pressure of carbon dioxide (PaCO_2_) during induction, an unrecognized hemoglobin decrease, or intense pain during emergence may be sufficient to precipitate ischemia in marginally perfused territories [20,21,22].

Neuromonitoring helps to achieve these goals. It is not, however, a substitute for hemodynamic management. For example, a clean somatosensory evoked potential during a period of unrecognized hyperventilation is not reassuring; an EEG change during deepening anesthesia should not be assumed to indicate ischemia. The most appropriate approach is to treat monitoring data as one input alongside MAP (mean arterial pressure), end-tidal CO_2_, oxygen saturation, hemoglobin, temperature, and the surgical step in progress, and to act on physiology rather than on a single monitoring signal in isolation [23,24].

This review presents a practical framework for EDAS anesthesia organized around five linked priorities: stable MAP at or modestly above baseline, deliberate avoidance of hypocapnia from hyperventilation, preservation of arterial oxygen content, neuromonitoring-informed intervention, and structured communication with the surgical team.

Several recent reviews already address the anesthetic management of moyamoya disease and converge on a shared core of advice: maintain a stable MAP, preserve normocapnia, avoid hyperventilation, and treat neuromonitoring as surveillance rather than as a stand-alone trigger for intervention [12,25,26]. This review does not dispute that consensus. Its intended contribution is narrower and more practical. First, it organizes management specifically around EDAS, an indirect procedure in which the operation confers no immediate intraoperative flow benefit, so that the entire anesthetic burden falls on protecting a pre-existing and marginal collateral circulation. Second, it foregrounds carbon dioxide management and the competing risks of hypocapnic vasoconstriction and hypercapnic intracerebral steal, a tension that prior reviews mention but rarely place at the center. Third, it offers an explicit, sequential intraoperative response algorithm intended for real-time use. The algorithm is presented as a proposed, expert-derived framework synthesizing physiologic reasoning and reported practice; it has not been prospectively validated in a clinical cohort, and the present work does not claim to validate it.

This is a narrative review rather than a systematic one. The supporting literature was identified through searches of PubMed/MEDLINE and Google Scholar covering the period from 1959 to 2026, combining the terms “moyamoya,” “encephaloduroarteriosynangiosis,” “EDAS,” “indirect revascularization,” “cerebral blood flow,” “cerebral autoregulation,” “carbon dioxide reactivity,” “intracerebral steal,” “anesthetic management,” and “neuromonitoring.” Priority was given to peer-reviewed studies, society guidelines, and primary physiologic sources, supplemented by foundational older references where they remain the original basis for a concept. Because the review is interpretive and not a formal evidence synthesis, no quantitative selection protocol, risk-of-bias assessment, or PRISMA flow was applied, and the resulting synthesis should be read as expert interpretation rather than as a comprehensive or reproducible search.

## 2. Pathophysiology Relevant to Anesthesia

### 2.1. Impaired Autoregulation and Pressure-Dependent Perfusion

In the healthy brain, cerebral blood flow (CBF) is maintained relatively constant over a wide range of mean arterial pressures because resistance vessels (arterioles) constrict or dilate to accommodate pressure changes [27,28]. In Moyamoya disease, that capacity has often already been spent. The distal arteriolar bed of an ischemic territory tends to be near-maximally dilated at rest, leaving little reserve to compensate for further drops in driving blood pressure. The classical autoregulation curve narrows, sometimes substantially, and within affected territories, CBF becomes functionally pressure-dependent [27,29,30].

The clinical implication is conceptually straightforward. Cerebral perfusion pressure, conventionally MAP minus intracranial pressure, is mostly determined by MAP during routine EDAS surgery, since intracranial pressure is rarely the dominant variable in an open craniotomy with normal compliance. This does not mean that higher is always better. It means that drops below the patient’s baseline MAP are more dangerous than they would be in a patient with intact autoregulation [10,20,22]. Practically, even modest reductions in MAP from baseline that an otherwise healthy patient would tolerate without symptoms may be associated with neurologic deterioration in patients with severe Moyamoya, with the magnitude of the tolerated drop varying by territory and individual patient [20,22]. Targets should be agreed upon with the surgical team and tailored to the individual patient.

### 2.2. Carbon Dioxide Reactivity and the Problem of Hyperventilation

Carbon dioxide is one of the most potent modulators of cerebral vascular tone, and in Moyamoya, it is also one of the most critical parameters to manage. Hypocapnia constricts cerebral resistance vessels and reduces CBF—that is the basic physiology that has made hyperventilation important for brain relaxation in tumor and trauma surgery for decades [31,32,33,34]. In a patient whose diseased territories have limited vasodilatory reserve, the same approach may further reduce regional cerebral perfusion.

Inadvertent hyperventilation may be subtle and can occur in situations such as a manually bagged induction with high minute ventilation, a controlled mode set with a respiratory rate or tidal volume that overshoots, or an emergence in which pain or anxiety increases the spontaneous respiratory rate. In Moyamoya, slight hypocapnia can produce measurable reductions in regional CBF and corresponding EEG changes [22,30,35].

Excessive hypercapnia should also not be assumed to be protective. Hypercapnia dilates cerebral vessels, but in heterogeneous cerebrovascular disease, the vessels that respond best are those with reserve—that is, the relatively normal ones. Maximally dilated, ischemic territories cannot dilate further. As CO_2_ rises, blood is drawn preferentially into healthier territories at the expense of compromised regions. This intracerebral steal phenomenon has been described in cerebrovascular disease since the 1960s and is plausibly relevant to Moyamoya, although the magnitude in any individual patient is unpredictable [32,33,34,36,37]. The safer ventilation target in EDAS is therefore neither hypocapnia nor permissive hypercapnia. It is controlled normocapnia, transitions made gradually rather than abruptly, and arterial confirmation of the EtCO_2_–PaCO_2_ gradient when the difference might matter.

A modest preference for the high end of normal EtCO_2_ is a reasonable individual choice in patients with severe disease, provided MAP and oxygenation are stable. This is not the same as deliberate hypercapnia and should not be presented as a generalizable recommendation.

**Figure 1:**
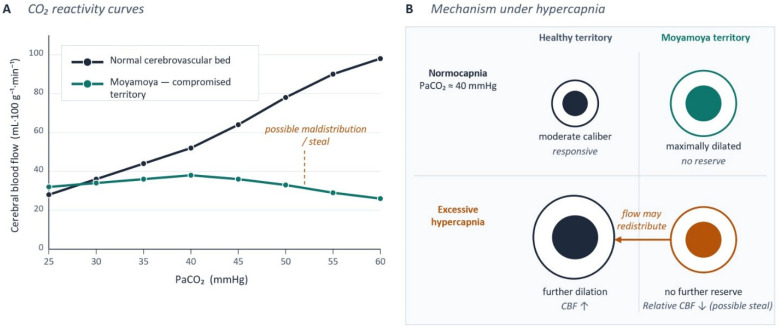
Cerebrovascular CO_2_ reactivity and potential intracerebral steal in moyamoya disease. Legend: (**A**) Schematic CO_2_ reactivity for a healthy cerebrovascular bed (black) and a moyamoya-affected territory (green). The moyamoya curve illustrates impaired reserve and the potential for regional maldistribution during excessive hypercapnia. (**B**) Mechanism. At normocapnia, healthy vessels retain dilatory reserve while moyamoya collaterals are near-maximally dilated. With excessive hypercapnia, healthy vessels can dilate further; the moyamoya bed cannot, and flow may be redistributed away from it (potential intracerebral steal). The schematic is conceptual; vessel dimensions are not to scale. The axes are illustrative and carry no specific data values; the curves indicate qualitative relationships only and should not be interpreted as measured reactivity data. Abbreviations: CBF, cerebral blood flow; CO_2_, carbon dioxide; PaCO_2_, arterial partial pressure of carbon dioxide.

The central vascular physiologic vulnerability during EDAS is summarized in Figure 1: impaired autoregulation makes cerebral blood flow more pressure-dependent, while deviations in PaCO_2_ may further compromise regional perfusion.

### 2.3. Oxygen Supply–Demand Balance

Beyond pressure and CO_2_, the anesthetic plan needs to maintain oxygen delivery and keep metabolic demand in check. Arterial oxygen saturation, hemoglobin concentration, and cardiac output together determine how much oxygen reaches tissue, and any of them can be disturbed during EDAS—by an unrecognized cuff leak, by surgical bleeding, by vasodilator overshoot, or by simple intravascular volume loss after a long fasting period. On the demand side, fever, seizure activity, pain, and shivering all increase cerebral metabolic rate. Maintaining adequate oxygen delivery while limiting metabolic demand is therefore as much a part of perfusion protection as pressure and CO_2_ control.

## 3. Preoperative Assessment and Planning

### 3.1. Disease Characterization

A useful preoperative evaluation for EDAS also takes into account the type of Moyamoya the patient has—ischemic-predominant, hemorrhagic, or mixed—the side of the brain and the extent of circulation involvement, the baseline neurologic examination, whether the patient has had prior strokes or seizures, and the habitual blood pressure [5,6,21,38]. Imaging review with the surgical team should clarify the donor vessel course, the planned site of synangiosis, and any territories considered most vulnerable. When perfusion reserve studies (acetazolamide-challenged SPECT, CT perfusion, or BOLD-CVR MRI) are available, they help identify regions where pressure dependence is likely to be most severe [21,30].

The agreed MAP range should be discussed with the surgery team. It is also worth asking the patient or family, especially in pediatric cases, about what is known to provoke symptoms. Crying, dehydration, hyperventilation during a tantrum, exertion in the heat, and prolonged fasting are common precipitants in children with Moyamoya [7,39,40]. In children with symptom provocation during crying or agitation, calm separation from caregivers and a smooth intravenous start may be especially important.

### 3.2. Hydration, Fasting, and Premedication

Prolonged fasting and dehydration shift cerebral physiology in the wrong direction by lowering preload and stroke volume [40,41]. Local fasting policies vary; in most centers, allowing clear liquids up to 2 h before induction and starting maintenance intravenous fluids the night before or on arrival is reasonable for patients with severe disease. Euvolemia is the preferred goal.

Premedication is a balance. Anxiety, agitation, and crying provoke hyperventilation and sympathetic instability, and are not neutral events in this disease [39,42]. On the other hand, oversedation can produce hypoventilation, hypoxia, or delayed neurologic assessment. Low-dose oral midazolam in cooperative children, with parental presence at induction where institutional culture permits, is a common approach. Because crying-induced hypocapnia is a specific and avoidable hazard in this population, several practical measures deserve emphasis. Oral midazolam (commonly about 0.5 mg/kg to a typical maximum near 20 mg) given 15–20 min before separation, or intranasal dexmedetomidine (approximately 1–2 mcg/kg) where available, can achieve calm separation without the respiratory depression that accompanies deeper sedation. Allowing a parent to be present at induction, minimizing waiting time, maintaining a quiet environment, and permitting a familiar comfort object all reduce the likelihood of a distress-driven respiratory surge. When intravenous access cannot be secured in an awake child without significant crying, an unhurried inhalational induction with sevoflurane in the parent’s arms or on the table—a calm, slow “steal” induction that avoids forcing a struggling, hyperventilating child—is often preferable to repeated distressing attempts at awake cannulation, with intravenous access then obtained promptly once anesthetized. The shared goal of each approach is to reach a controlled airway and stable ventilation before hypocapnia or a sympathetic surge can develop. Adults often need less. The aim is calm transfer to the operating room and smooth induction while avoiding excessive preoperative sedation.

## 4. Intraoperative Hemodynamic Management

The single most consistent intraoperative objective in EDAS is maintaining a stable MAP. Many practices target baseline or slightly above—often within roughly 10–20% above the patient’s usual pressure when ischemic risk is high—although no high-quality trial defines a single correct number, and individualization based on disease severity, baseline hypertension, surgical step, and surgeon preference remains appropriate [12,20,25,26].

Particular attention should be paid to avoiding hypotension during transitions such as induction, airway manipulation, head positioning and pinning, skin incision, donor vessel dissection, dural opening, emergence, and transfer from the operating table to the bed or ICU stretcher [20,21,22]. A practical safeguard is to have a vasopressor prepared and immediately available before induction begins, particularly in patients at high ischemic risk. When disease severity is high or hemodynamic lability is anticipated, initiating a low-rate infusion of phenylephrine or norepinephrine before induction is reasonable [12,14].

An arterial line is strongly favored. Intermittent oscillometric measurements miss the very brief but substantial pressure drops that EDAS patients tolerate poorly, and an arterial waveform also provides early warning of hypovolemia and stroke volume variation [13,26]. Whether the line is placed before or after induction depends on the patient. As an illustration in a single reported EDAS case managed by the author, a radial arterial line placed before induction allowed continuous blood-pressure monitoring and serial hemoglobin measurement by arterial blood gas analysis while strict normotension and normocapnia were maintained; electroencephalographic monitoring was not employed in that case [43]. Whatever the choice, the transducer should be zeroed at a consistent reference (typically the tragus or the level of the right atrium, with the choice declared explicitly), and the agreed MAP target should be visible on the monitor and known to anesthesia, surgery, and neuromonitoring teams.

Phenylephrine has the advantages of familiarity, predictability, and rapid titration through a peripheral line for short interventions. Norepinephrine is a reasonable choice when an infusion is anticipated, when cardiac output support is also wanted, or when persistent vasoconstriction with phenylephrine is a concern. Ephedrine is less predictable and may be useful when bradycardia accompanies hypotension, but it should not be the default. The literature does not establish a single agent as superior for Moyamoya disease specifically [12,26], and the practical objective is prompt correction of hypotension while minimizing recurrent or prolonged blood pressure deviations.

Balanced crystalloid is the usual maintenance fluid; institutional practice varies on the use of colloids and on volume targets. Hypovolemia clearly worsens cerebral perfusion. Aggressive hypervolemia does not reliably help and may complicate postoperative management. Hemoglobin should be kept at a level that supports oxygen delivery rather than at a fixed transfusion trigger borrowed from an unrelated population; a higher threshold is reasonable in patients with severe perfusion compromise [20,26,44].

## 5. Ventilation: Avoiding Hyperventilation and Maintaining Normocapnia

Ventilation deserves focused discussion because PaCO_2_ is a highly modifiable determinant of cerebral vascular tone during EDAS, and deviations may have immediate effects on regional cerebral perfusion.

The rationale for avoiding hyperventilation is central to the anesthetic management of EDAS. The diseased vascular bed in Moyamoya is, in many territories, near-maximally dilated [10,30]. Hypocapnia constricts cerebral resistance vessels, including, to whatever extent they are still capable of constricting, the diseased ones. Even a transient PaCO_2_ of 28–30 mmHg can reduce regional CBF in territories without compensatory reserve [22,32]. Routine hyperventilation is generally difficult to justify during EDAS and should be avoided unless a specific, time-limited surgical indication is identified. The traditional indication for hypocapnia in neurosurgery is rarely needed for EDAS—the procedure does not require deep retraction of the swollen brain. If the surgeon does request transient relaxation, it should be discussed explicitly, time-limited, and monitored.

The argument against routine intentional hypercapnia is more nuanced but equally clinically important. The intuitive appeal is obvious: if hypocapnia causes vasoconstriction, then higher CO_2_ should increase flow. The limitation is that the regional distribution of increased flow cannot be reliably controlled. Hypercapnia dilates vessels that can still dilate, which preferentially means those supplying relatively healthy territories. In a heterogeneously diseased brain, blood is therefore drawn away from the maximally dilated, ischemia-prone territories that need it most. This intracerebral steal phenomenon has been described in cerebrovascular disease since the 1960s and is plausibly relevant to Moyamoya, although the magnitude in any individual patient is unpredictable [32,33,34,36,37]. Intentional hypercapnia may therefore introduce competing risks, particularly when regional cerebrovascular reserve is heterogeneous.

For most patients, the appropriate target is a steady PaCO_2_ in the high-30 s, with EtCO_2_ adjusted accordingly and abrupt transitions avoided [29,31]. Early in the case, arterial blood gas confirmation is useful because the EtCO_2_–PaCO_2_ gradient varies with dead space, ventilation–perfusion mismatch, and cardiac output, and a given EtCO_2_ value may correspond to a meaningfully higher PaCO_2_, with the gradient widening when these factors are altered. Coughing, agitation, light anesthesia, and pain during emergence all alter ventilation and CO_2_ in ways that may not be appreciated until EEG changes occur. The principle is to treat the trigger, not the number.

The position taken in this review is that hyperventilation should be avoided, normocapnia is the default target, mild high-normal CO_2_ may be acceptable in selected patients with stable MAP and oxygenation, and routine intentional hypercapnia is not recommended.

The primary physiologic targets during EDAS anesthesia are summarized in Table 1.

## 6. Anesthetic Technique

### 6.1. Induction

Induction should be smooth, slow enough to allow continuous pressure correction, and free of coughing, hyperventilation, hypoxia, and sympathetic surges [12,14,39]. The choice of agent matters less than how it is administered. Propofol with a moderate-dose opioid and a neuromuscular blocker suitable for the planned monitoring is a reasonable default for adults; etomidate is an alternative when hemodynamic stability is a particular concern, despite its known endocrine and neurologic considerations. In children, an inhalational induction may be unavoidable, in which case calm parental presence, attention to spontaneous ventilation, and prompt intravenous access for rescue agents are central [39,42,45,46]. In higher-risk patients, vasopressors should be immediately available before induction.

### 6.2. Maintenance

Both volatile-based and total intravenous techniques have been used successfully in EDAS [12,13,14,25,35]. A retrospective comparison of sevoflurane and propofol maintenance in adult moyamoya revascularization found no significant difference in postoperative stroke or transient neurological deficits between the two, although intravenous anesthesia was associated with lower intraoperative blood-pressure variability [47]. Total intravenous anesthesia (TIVA) with propofol and remifentanil can simplify the interpretation of evoked potentials and provide a steady plane of anesthesia that responds quickly to titration. Volatile maintenance at modest age-adjusted MAC is compatible with raw EEG-based ischemia surveillance and remains the routine choice in many centers. The decision should be driven by planned monitoring, the surgeon’s expected operative time, and local familiarity rather than by ideology. Whichever technique is chosen, the priorities are: stable MAP, normocapnia, normoxia, normothermia, and anesthetic depth sufficient to prevent movement and sympathetic responses without causing EEG suppression that would confound interpretation. Practical EEG use may also be constrained by surgical draping, positioning, or access to the forehead. Alternative frontal electrode configurations have been described to facilitate EEG monitoring when standard placement is limited by procedural constraints [48].

### 6.3. Analgesia and Emergence

Pain after EDAS is typically scalp-derived and responds to a balanced regimen of an intermediate-acting opioid, paracetamol, and—where institutional practice permits—scalp infiltration with local anesthetic at closure. Emergence is the second high-risk transition. Coughing, bucking, hypertensive surges, and pain-driven hyperventilation may be particularly hazardous during emergence, when monitoring intensity and surgical stimulation are changing. Lidocaine, low-dose dexmedetomidine, and titrated remifentanil have all been used to smooth emergence [12,14]. Representative adult dose ranges reported for this purpose include intravenous lidocaine at approximately 1.0–1.5 mg/kg given before extubation, a low-dose dexmedetomidine bolus of approximately 0.3–0.5 mcg/kg (often over 10 min to limit hypotension and bradycardia), or a low-rate remifentanil infusion of approximately 0.02–0.05 mcg/kg/min titrated to suppress coughing while preserving spontaneous ventilation. These values are illustrative starting points rather than disease-specific targets; doses should be reduced and individualized in children, in older adults, and whenever hemodynamic stability is the overriding concern; the unifying point is that emergence should be planned with the same care as induction.

## 7. Neuromonitoring During EDAS

### 7.1. The Conceptual Role

Neuromonitoring during EDAS is best understood as ischemia surveillance, interpreted alongside systemic physiology [23,24]. It is not a substitute for hemodynamic management. Any modality can be misleading in isolation, and any meaningful change should prompt a coordinated review of MAP, ventilation, oxygenation, hemoglobin, anesthetic depth, temperature, and the surgical step in progress before being accepted as a primary signal of ischemia.

The principal neuromonitoring modalities relevant to EDAS and Moyamoya revascularization are summarized in Table 2.

### 7.2. Raw Electroencephalography

Raw EEG is a widely used electrophysiologic monitor for EDAS in many centers [26,40,42,49]. Continuous bilateral cortical activity, displayed in a format the team is trained to interpret, provides a near-real-time view of the population activity of the cortex under and adjacent to the surgical field. The changes that raise alarm include new focal slowing, loss of faster frequencies, regional attenuation, or asymmetry developing in temporal association with hypotension, ventilation changes, surgical manipulation, or anesthetic boluses. EEG is sensitive to anesthetic depth, temperature, baseline abnormalities, age, and artifact, so changes should trigger evaluation, not reflex action [24].

Processed and quantitative EEG indices are not emphasized here. These indices summarize complex tracings into single numbers, which can obscure the focal and asymmetric findings most informative in EDAS, and their thresholds for ischemia have not been established for this population. Raw waveform interpretation by an experienced team remains the primary modality.

### 7.3. Adjunctive Electrophysiologic Monitoring

Somatosensory evoked potentials (SSEPs) assess the dorsal column–medial lemniscal pathway from the peripheral nerve to the somatosensory cortex and can detect regional ischemia affecting these structures. Warning criteria—typically a 50% amplitude decrease or 10% latency prolongation, though institutional thresholds vary—should be agreed upon in advance with the neurophysiology team [23,24,50]. SSEP is anesthetic-sensitive and can be confounded by temperature, hemoglobin, and technical factors, so it should not be the sole source of information [24,50].

Motor evoked potentials (MEPs) provide pathway-specific information about the corticospinal tract and may detect motor pathway compromise that SSEP misses [51,52]. Their use imposes constraints on the anesthetic technique—including limited or no neuromuscular blockade after intubation, attention to bite blocks, and TIVA in many centers—and is generally reserved for cases in which motor pathway risk is judged high. As with SSEP, MEP in EDAS should be presented as an option used in selected centers rather than a universal standard.

### 7.4. Near-Infrared Spectroscopy and Doppler Tools

Near-infrared spectroscopy (NIRS) provides a noninvasive, regional estimate of frontal cortical oxygenation [53,54]. It is most useful as a trend monitor—an unexpected fall during induction, positioning, or after a vasodilator bolus prompts evaluation—rather than as a single threshold-driven indicator. Well-recognized limitations include extracranial signal contamination, limited spatial resolution, and uncertain absolute thresholds for intervention [53,54,55,56]. NIRS may complement EEG and hemodynamic monitoring, but should not be the sole determinant of management. A particular caveat applies to the intracerebral steal phenomenon illustrated in Figure 1. The available intraoperative monitors are not well suited to detecting a regional steal event with confidence. NIRS interrogates a small volume of frontal cortex immediately beneath the sensors and reports surface oxygenation only, so redistribution of flow away from a deep or non-frontal territory may not register at all. Scalp EEG is similarly weighted toward cortical convexity activity and may be insensitive to ischemia confined to deep or sub-cortical regions. Both modalities also sample a limited area relative to the heterogeneous, patchy distribution of moyamoya vasculopathy. The practical implication is that a reassuring NIRS or EEG trace does not exclude regional hypoperfusion from steal, and the case against deliberate hypercapnia therefore rests primarily on physiologic reasoning rather than on a monitor capable of reliably detecting the event in real time.

Transcranial Doppler can provide flow-velocity information in selected centers with the necessary expertise; intraoperative use during EDAS is uncommon. Handheld Doppler assessment of donor vessel patency at the end of the procedure is a separate, surgically directed tool used in some centers [49].

Table 2 reflects the working position of this review: raw EEG and stable hemodynamics at the core; SSEP, MEP, NIRS, and TCD added when local expertise and patient factors justify them; and no claim that any single multimodal package represents a universal standard.

## 8. Integrated Intraoperative Response Algorithm

A suspected ischemic event during EDAS may present as new EEG slowing, deterioration of evoked potential responses, NIRS desaturation, an unexpected drop in MAP, an EtCO_2_ deviation, or a concern raised by the surgical team. The response should be immediate, structured, and communicated.

The first step is to verify the signal. Loose electrodes, cautery artifact, irrigation cooling, head movement during pinning, or a recent anesthetic bolus can all produce changes that mimic ischemia [24]. A few seconds spent confirming that the signal is real prevents reflexive interventions that may themselves cause harm.

The second step is to correct perfusion drivers. MAP should be restored to baseline or slightly above with a vasopressor bolus, while volume status, blood loss, and recent anesthetic depth are evaluated [10,20,22]. If a phenylephrine bolus does not promptly restore pressure, volume loss, ongoing vasodilation, or inadequate preload should be considered, and fluid administration or vasopressor infusion may be required.

The third step is to correct ventilation. If hyperventilation is present, it should be stopped, and the EtCO_2_ allowed to drift back into the high-30 s rather than overshooting into hypercapnia [31,32]. Abrupt changes in CO_2_ are themselves a perturbation. An arterial blood gas confirms the working number when the gradient is uncertain.

The fourth step is to optimize oxygen delivery. SpO_2_, FiO_2_, hemoglobin, and temperature should be confirmed; transfusion should be considered if bleeding has exceeded estimates; warming should be optimized if drift has occurred.

The fifth step is to assess anesthetic depth. Excessive depth suppresses EEG activity and can mimic ischemic slowing; inadequate depth permits sympathetic surges and movement [24,50]. Either error confounds the picture.

The sixth step is to communicate with the surgeon. Surgical contributors such as mechanical compression, scalp flap pressure, donor vessel kinking, retractor effect, or positioning should be considered. The conversation should be brief, specific, and bilateral.

After intervention, the team should reassess. If the change does not resolve after perfusion, ventilation, oxygenation, and anesthetic depth have been addressed and the surgical field has been reviewed, escalation—including imaging, ICU disposition with continued monitoring, and senior surgical consultation—is warranted.

**Figure 2:**
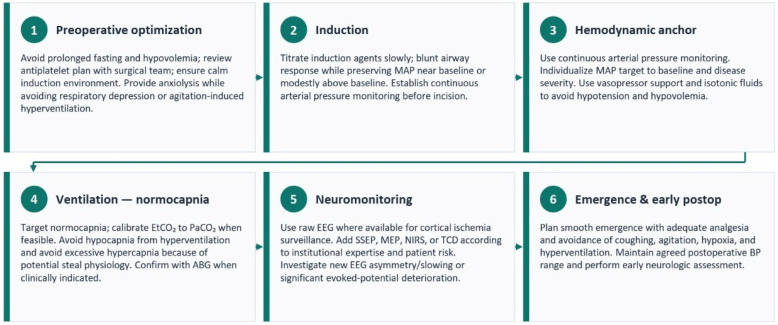
Six-step intraoperative anesthesia algorithm for encephaloduroarteriosynangiosis (EDAS). Legend: Sequential phases of intraoperative care for EDAS in moyamoya disease. The underlying physiologic goal across all six phases is to maintain regional cerebral perfusion: avoid hypotension, avoid hypocapnia, and avoid excessive hypercapnia. Specific drug choices, vasopressor selection, and monitoring intensity should be individualized to the patient and to local practice. Abbreviations: ABG, arterial blood gas; BP, blood pressure; EDAS, encephaloduroarteriosynangiosis; EEG, electroencephalography; EtCO_2_, end-tidal carbon dioxide; MAP, mean arterial pressure; MEP, motor evoked potential; NIRS, near-infrared spectroscopy; PaCO_2_, arterial partial pressure of carbon dioxide; SSEP, somatosensory evoked potential; TCD, transcranial Doppler.

A structured response to suspected intraoperative hypoperfusion during EDAS is shown in Figure 2. The description below is intended for practical intraoperative use.

## 9. Postoperative Management

EDAS patients remain at risk during the recovery phase. Hypoperfusion from postoperative hypotension, dehydration, or pain-driven sympathetic instability is the more common concern [20,44,57]. Hyperperfusion syndrome is classically described after direct bypass [58] but has been reported in mixed series after indirect procedures and should not be dismissed [59,60,61]. Seizures, postoperative pain, fever, nausea-driven vomiting, and ventilation abnormalities can each cause or mimic neurologic deterioration.

Early neurologic examination is valuable, but extubation should be unhurried. The patient who is hemodynamically stable, normocapnic on spontaneous ventilation, normothermic, and comfortable will tolerate emergence and extubation; the patient who meets airway criteria but remains hypertensive from inadequate analgesia or hypocapnic from anxiety may be at increased risk of postoperative ischemic deterioration. Emergence agitation and post-anesthesia care unit delirium are relevant concerns in this population: in a large pediatric series undergoing indirect revascularization, delirium in the recovery unit was common and was independently associated with greater intraoperative blood-pressure variability, while total intravenous anesthesia appeared protective [62]. Postoperative priorities are, in essence, the continuation of intraoperative priorities: avoid hypotension, avoid dehydration, maintain normoxia and normocapnia, provide adequate analgesia and antiemesis, treat fever, watch for seizures, and keep the agreed blood pressure range visible to the postoperative team.

The transfer from the operating room to the PACU or ICU deserves specific attention, because it is an interval in which monitoring is often interrupted just as the patient is most vulnerable to undetected hemodynamic change. The same physiologic priorities that govern intraoperative care do not pause during transport. Continuous monitoring of blood pressure and oxygenation should be maintained throughout the move rather than resumed only on arrival, since a brief, unobserved episode of hypotension or hypoxia in transit can precipitate ischemia in a marginally perfused territory before it is recognized. Practically, this means transferring the patient with continued pulse oximetry and blood-pressure monitoring, keeping vasopressor and the agreed MAP target immediately available en route, ensuring adequate analgesia and a calm transfer to avoid pain- or anxiety-driven hyperventilation, and giving a structured handover that communicates the target blood-pressure range to the receiving team before monitoring is handed over.

Differentiating hypoperfusion from hyperperfusion, postoperative seizure, edema, or vascular complications often requires imaging and is rarely possible on clinical grounds alone [59,60,61]. A low threshold for CT, perfusion imaging, or MRI is appropriate in any patient with new neurologic findings.

## 10. Discussion

Anesthetic management for EDAS differs from other neurosurgical anesthesia because the operation does not alter cerebral physiology on the day of surgery. Synangiosis matures slowly [19]. During the operation, cerebral perfusion remains dependent on the patient’s pre-existing collateral network, with impaired autoregulation and limited vasodilatory reserve still present. The anesthetic plan therefore protects what is already there rather than facilitating something new.

The thread that runs through this review is integration. MAP, PaCO_2_, oxygenation, hemoglobin, temperature, anesthetic depth, surgical step, and neuromonitoring are not independent variables. New EEG slowing during unrecognized hypotension and inadvertent hyperventilation is a different event from EEG slowing after a propofol bolus, and the response differs [24]. A stable SSEP during hyperventilation is not proof that the brain is well perfused; it is proof that the somatosensory pathway being monitored is still functioning, which is not the same thing [23,50]. Effective EDAS anesthesia requires the team to interpret these signals together rather than separately.

The argument against routine hyperventilation warrants restatement because the practice has historical inertia. In tumor and trauma neurosurgery, hyperventilation has been used for decades to reduce brain bulk [32,33]. EDAS is fundamentally different: the cortex does not need to be retracted, the brain does not need to be relaxed, and the diseased vascular bed cannot tolerate the vasoconstrictive cost [10,20,22]. Normocapnia is the safer default, and hyperventilation, when it occurs, is more often inadvertent than intentional. Vigilance against drift is part of the anesthetic.

Also, the argument against routine intentional hypercapnia is more recent and less widely accepted. The reasoning—that a diseased vascular bed cannot dilate further while healthier territories can, producing intracerebral steal—rests on physiologic reasoning together with limited empirical data, including a small intraoperative series in moyamoya in which falls in regional cerebral blood flow accompanied global hyperemia [32,34,37]. The clinical literature on this question is limited, and practice clearly varies. The position taken here is conservative: tolerate the high end of normal in selected patients, but do not pursue hypercapnia as a goal.

Neuromonitoring practice in EDAS is institution-dependent and likely to remain so [26,49]. Raw EEG is widely used and well-suited for surveillance of cortical ischemia. SSEP, MEP, NIRS, and TCD provide complementary information but require local expertise for accurate interpretation. A review that overstates a single modality risks losing relevance for centers that practice differently; a review that ignores adjuncts risks being incomplete. The structure of Table 2 reflects an attempt to acknowledge both realities.

Several limitations of this review warrant mention. The literature specific to EDAS anesthesia is largely descriptive, with single-center series, retrospective cohorts, and expert reviews dominating [12,14,25,26]. Randomized trials evaluating vasopressor choice, specific MAP targets, or ventilation strategies in this population are lacking. The framework presented here is therefore a synthesis of physiologic reasoning and reported practice, not a guideline derived from comparative evidence. It is worth being explicit about how strongly each recommendation is supported. The avoidance of hypocapnia rests on well-established CO_2_ reactivity physiology together with observational moyamoya data showing reduced regional flow during hyperventilation, and is the most secure of the recommendations. The preference for a stable MAP at or modestly above baseline is supported by observational series linking intraoperative hypotension to ischemic complications, but no trial defines a single correct target, so the specific 10–20% figure reflects common practice rather than comparative evidence. The caution against deliberate hypercapnia is the weakest-supported position: it rests largely on the steal concept and a small intraoperative series, and is offered as prudent reasoning rather than as established fact. Recommendations on vasopressor choice, fluid strategy, transfusion thresholds, and the specific composition of a neuromonitoring package are similarly grounded in physiologic rationale and reported practice rather than in controlled comparison. Centers may reasonably differ in their thresholds, monitoring choices, and emergence strategies. The principles—perfusion preservation, controlled normocapnia, oxygen delivery, neuromonitoring-informed intervention, and structured communication—are likely to be more durable than any specific number.

A word is warranted on the status of the proposed six-step algorithm. Its individual components are not themselves novel; in the author’s experience they reflect routine EDAS practice, in which the electrophysiologic monitoring element has been raw EEG, supplemented by SSEP. This experience includes a single-center retrospective review of 15 adults undergoing EDAS, reported in abstract form, in which intraoperative EEG and SSEP monitoring were used in every case and recordings remained stable throughout, with no intraoperative EEG changes and favorable neurologic outcomes [63]. What has been lacking is not the individual elements but an explicit framework that organizes these established components into a single sequential intraoperative workflow. The algorithm is therefore offered as a structured synthesis of existing practice and expert reasoning rather than as a novel or prospectively validated protocol; it has not been tested against an alternative approach in a controlled fashion, and prospective evaluation would be required before it could be presented as more than a pragmatic framework.

## 11. Conclusions

EDAS anesthesia is best understood as a perfusion-preservation strategy for a brain with limited autoregulatory reserve and collaterals that will not be reinforced for weeks to months. The central goals are maintaining stable MAP at or modestly above baseline, deliberately avoiding hypocapnia from hyperventilation, preserving arterial oxygen content, and integrating neuromonitoring with systemic physiology. Normocapnia is the default ventilation target. Routine intentional hypercapnia is not recommended because of the risk of intracerebral steal, although the high end of the normal range may be tolerated in individual patients.

Where it is available and the team is experienced in its interpretation, raw EEG is a useful modality for cortical ischemia surveillance during EDAS, with SSEP, MEP, NIRS, and TCD added when local expertise and patient factors justify their use. The strength of this recommendation is necessarily tempered by the reality that neuromonitoring practice is institution-dependent and that EEG access can be constrained by the surgical setup, so no single modality should be presented as a universal standard. In the author’s institutional EDAS experience, combined EEG and SSEP monitoring was applied in every case and remained stable intraoperatively [63]. The structured response algorithm presented here—verify the signal, correct perfusion, correct ventilation, optimize oxygen delivery, review anesthetic depth, and communicate with the surgeon—is intended as a practical framework for the operating room rather than a definitive guideline. In a procedure whose surgical benefit is delayed by weeks, intraoperative anesthetic management is critical because cerebral perfusion remains dependent on pre-existing collateral circulation until the new collateral network matures.

## Figures and Tables

**Table 1:** Physiologic targets during EDAS.

Variable	Preferred Target	Rationale
MAP	Baseline or modestly above baseline	Preserves pressure-dependent CBF in territories with exhausted vasodilator reserve
PaCO_2_	Normocapnia (~35–40 mmHg)	Avoids hypocapnic vasoconstriction and possible hypercapnic intracerebral steal
Oxygenation	Normoxia/high-normal SpO_2_	Preserves arterial oxygen content in a brain with limited flow reserve
Hemoglobin	Avoid clinically significant anemia	Supports oxygen delivery; threshold individualized to disease severity
Temperature	Normothermia	Avoids increased cerebral metabolic demand from fever
Volume	Euvolemia	Avoids hypoperfusion from hypovolemia without unnecessary overload

Abbreviations: CBF, cerebral blood flow; EDAS, encephaloduroarteriosynangiosis; MAP, mean arterial pressure; PaCO_2_, arterial partial pressure of carbon dioxide; SpO_2_, peripheral oxygen saturation.

**Table 2:** Neuromonitoring modalities in EDAS and Moyamoya revascularization.

Modality	Physiologic Domain	Strength	Typical Ischemic Warning Criteria	Limitation	Positioning
Raw EEG	Cortical activity	Continuous cortical ischemia surveillance	New focal slowing, loss of fast (beta) activity, regional attenuation, or new asymmetry	Anesthetic and artifact sensitivity	Commonly used where available; practice varies by center
SSEP	Sensory pathway	Pathway-specific data complementary to the EEG	≈50% amplitude decrease or ≈10% latency increase (institution-dependent)	Anesthetic-sensitive; delayed change	Adjunct in selected centers
MEP	Motor pathway	Detects corticospinal compromise not seen on SSEP	Marked amplitude reduction or loss of response (threshold/criteria vary by center)	Requires limited neuromuscular blockade and TIVA in many centers	Adjunct in selected centers
NIRS	Regional oxygenation	Noninvasive trend monitor	Sustained fall in regional saturation from baseline (commonly cited as ≈>20%; no validated threshold)	Extracranial contamination; uncertain thresholds	Optional adjunct
TCD/Doppler	Flow velocity/vessel patency	Flow-related information: donor vessel check	Substantial change in flow velocity or loss of donor-vessel signal (qualitative)	Operator-dependent; not universal in EDAS	Context-dependent adjunct

Abbreviations: EDAS, encephaloduroarteriosynangiosis; EEG, electroencephalography; MEP, motor evoked potential; NIRS, near-infrared spectroscopy; SSEP, somatosensory evoked potential; TCD, transcranial Doppler; TIVA, total intravenous anesthesia.

## Data Availability

No new data were created or analyzed in this study. Data sharing is not applicable to this article.

## References

[1] Suzuki J., Takaku A. (1969). Cerebrovascular “moyamoya” disease: Disease showing abnormal net-like vessels in base of brain. Arch. Neurol..

[2] Hayashi K., Horie N., Suyama K., Nagata I. (2013). An epidemiological survey of moyamoya disease, unilateral moyamoya disease, and quasi-moyamoya disease in Japan. Clin. Neurol. Neurosurg..

[3] Scott R.M., Smith E.R. (2009). Moyamoya disease and moyamoya syndrome. N. Engl. J. Med..

[4] Kuroda S., Houkin K. (2008). Moyamoya disease: Current concepts and future perspectives. Lancet Neurol..

[5] (2012). Research Committee on the Pathology and Treatment of Spontaneous Occlusion of the Circle of Willis; Health Labour Sciences Research Grant for Research on Measures for Intractable Diseases. Guidelines for diagnosis and treatment of moyamoya disease (spontaneous occlusion of the circle of Willis). Neurol. Med. Chir..

[6] Sato Y., Kazumata K., Nakatani E., Houkin K., Kanatani Y. (2019). Characteristics of moyamoya disease based on national registry data in Japan. Stroke.

[7] Takanashi J. (2011). Moyamoya disease in children. Brain Dev..

[8] Roach E.S., Golomb M.R., Adams R., Biller J., Daniels S., Deveber G., Ferriero D., Jones B.V., Kirkham F.J., Scott R.M. (2008). Management of stroke in infants and children: A scientific statement from a Special Writing Group of the American Heart Association Stroke Council and the Council on Cardiovascular Disease in the Young. Stroke.

[9] Imaizumi T., Hayashi K., Saito K., Osawa M., Fukuyama Y. (1998). Long-term outcomes of pediatric moyamoya disease monitored to adulthood. Pediatr. Neurol..

[10] Smith E.R., Scott R.M. (2005). Surgical management of moyamoya syndrome. Skull Base.

[11] Macyszyn L., Attiah M., Ma T.S., Ali Z., Faught R., Hossain A., Man K., Patel H., Sobota R., Zager E.L. (2017). Direct versus indirect revascularization procedures for moyamoya disease: A comparative effectiveness study. J. Neurosurg..

[12] Parray T., Martin T.W., Siddiqui S. (2011). Moyamoya disease: A review of the disease and anesthetic management. J. Neurosurg. Anesthesiol..

[13] Chui J., Manninen P., Sacho R.H., Venkatraghavan L. (2015). Anesthetic management of patients undergoing intracranial bypass procedures. Anesth. Analg..

[14] Giustini A.J., Stone S.A., Ramamoorthy C. (2020). Moyamoya disease in children and its anesthetic implications: A review. Paediatr. Anaesth..

[15] Karasawa J., Kikuchi H., Furuse S., Sakaki T., Yoshida Y., Ohnishi H., Taki W. (1977). A surgical treatment of “moyamoya” disease: “Encephalo-myo synangiosis”. Neurol. Med. Chir..

[16] Matsushima T., Inoue T., Suzuki S.O., Fujii K., Fukui M., Hasuo K. (1992). Surgical treatment of moyamoya disease in pediatric patients—Comparison between the results of indirect and direct revascularization procedures. Neurosurgery.

[17] Pandey P., Steinberg G.K. (2011). Neurosurgical advances in the treatment of moyamoya disease. Stroke.

[18] Sun H., Wilson C., Ozpinar A., Safavi-Abbasi S., Zhao Y., Nakaji P., Wanebo J.E., Spetzler R.F. (2016). Perioperative complications and long-term outcomes after bypasses in adults with moyamoya disease: A systematic review and meta-analysis. World Neurosurg..

[19] Houkin K., Kuroda S., Ishikawa T., Abe H. (2000). Neovascularization (angiogenesis) after revascularization in moyamoya disease: Which technique is most useful for moyamoya disease?. Acta Neurochir..

[20] Sakamoto T., Kawaguchi M., Kurehara K., Kitaguchi K., Furuya H., Karasawa J. (1997). Risk factors for neurologic deterioration after revascularization surgery in patients with moyamoya disease. Anesth. Analg..

[21] Kim J.S. (2016). Moyamoya disease: Epidemiology, clinical features, and diagnosis. J. Stroke.

[22] Iwama T., Hashimoto N., Yonekawa Y. (1996). The relevance of hemodynamic factors to perioperative ischemic complications in childhood moyamoya disease. Neurosurgery.

[23] Nuwer M.R., Dawson E.G., Carlson L.G., Kanim L.E., Sherman J.E. (1995). Somatosensory evoked potential spinal cord monitoring reduces neurologic deficits after scoliosis surgery: Results of a large multicenter survey. Electroencephalogr. Clin. Neurophysiol..

[24] Sloan T.B. (1998). Anesthetic effects on electrophysiologic recordings. J. Clin. Neurophysiol..

[25] Yang K.J., Mistry P., Ayrian E. (2024). Update on the anesthesia management in adult patients with moyamoya disease. Curr. Opin. Anaesthesiol..

[26] Williams G.W., Jones W.S., Chaudhry R., Cai C., Pednekar G.S., Long A.C., Chouhan S., Artime C., Wegner R.C., Grewal N.K. (2019). Intraoperative anesthesiology management and patient outcomes for surgical revascularization for moyamoya disease: A review and clinical experience. J. Neurol. Surg. A Cent. Eur. Neurosurg..

[27] Lassen N.A. (1959). Cerebral blood flow and oxygen consumption in man. Physiol. Rev..

[28] Paulson O.B., Strandgaard S., Edvinsson L. (1990). Cerebral autoregulation. Cerebrovasc. Brain Metab. Rev..

[29] Willie C.K., Tzeng Y.C., Fisher J.A., Ainslie P.N. (2014). Integrative regulation of human brain blood flow. J. Physiol..

[30] Kuwabara Y., Ichiya Y., Otsuka M., Tahara T., Gunasekera R., Hasuo K., Masuda K., Matsushima T., Fukui M. (1990). Cerebral hemodynamic change in the child and the adult with moyamoya disease. Stroke.

[31] Meng L., Gelb A.W. (2015). Regulation of cerebral autoregulation by carbon dioxide. Anesthesiology.

[32] Brian J.E. (1998). Carbon dioxide and the cerebral circulation. Anesthesiology.

[33] Kety S.S., Schmidt C.F. (1948). The effects of altered arterial tensions of carbon dioxide and oxygen on cerebral blood flow and cerebral oxygen consumption of normal young men. J. Clin. Investig..

[34] Reivich M. (1964). Arterial PCO_2_ and cerebral hemodynamics. Am. J. Physiol..

[35] Sato K., Shirane R., Kato M., Yoshimoto T. (1999). Effect of inhalational anesthesia on cerebral circulation in moyamoya disease. J. Neurosurg. Anesthesiol..

[36] Symon L. (1969). The concept of intracerebral steal. Int. Anesthesiol. Clin..

[37] Oshima H., Katayama Y., Hirayama T. (2000). Intracerebral steal phenomenon associated with global hyperemia in moyamoya disease during revascularization surgery. J. Neurosurg..

[38] Kraemer M., Heienbrok W., Berlit P. (2008). Moyamoya disease in Europeans. Stroke.

[39] Soriano S.G., Sethna N.F., Scott R.M. (1993). Anesthetic management of children with moyamoya syndrome. Anesth. Analg..

[40] Nomura S., Kashiwagi S., Uetsuka S., Uchida T., Kubota H., Ito H. (2001). Perioperative management protocols for children with moyamoya disease. Child’s Nerv. Syst..

[41] Sato K., Shirane R., Yoshimoto T. (1997). Perioperative factors related to the development of ischemic complications in patients with moyamoya disease. Child’s Nerv. Syst..

[42] Kansha M., Irita K., Takahashi S., Matsushima T. (1997). Anesthetic management of children with moyamoya disease. Clin. Neurol. Neurosurg..

[43] Chauhan V., Sexton A. (2026). Anesthetic management of a patient with factor XIII deficiency undergoing encephaloduroarteriosynangiosis (EDAS): A case report and literature review. Cureus.

[44] Hyun S.J., Kim J.S., Hong S.C. (2010). Prognostic factors associated with perioperative ischemic complications in adult-onset moyamoya disease. Acta Neurochir..

[45] Brown S.C., Lam A.M. (1987). Moyamoya disease—A review of clinical experience and anaesthetic management. Can. J. Anaesth..

[46] Sumikawa K., Nagai H. (1983). Moyamoya disease and anesthesia. Anesthesiology.

[47] Cheng Y., Zha C., Che X., Wang Y. (2025). Inhalational versus intravenous anesthetic for cerebrovascular accident outcomes after surgical revascularization for adult moyamoya disease. BMC Anesthesiol..

[48] Isik O.G., Chauhan V., Ahmed M.T., Chang B.A., Cassim T.Z., Graves M.C., Rajan S., Garcia P.S. (2025). Alternate electrode placements to facilitate frontal electroencephalography monitoring in anesthetized and critically ill patients. J. Neurosurg. Anesthesiol..

[49] Chan J.L., Quintero-Consuegra M.D., Babadjouni R.M., Chang D., Barnard Z.R., Martin N.A., Ziv K., Van de Wiele B.M., Gonzalez N.R. (2022). Encephaloduroarteriosynangiosis operative technique and intraoperative anesthesia management: Treatment from both sides of the curtain. Oper. Neurosurg..

[50] Banoub M., Tetzlaff J.E., Schubert A. (2003). Pharmacologic and physiologic influences affecting sensory evoked potentials: Implications for perioperative monitoring. Anesthesiology.

[51] MacDonald D.B. (2006). Intraoperative motor evoked potential monitoring: Overview and update. J. Clin. Monit. Comput..

[52] Sloan T.B., Heyer E.J. (2002). Anesthesia for intraoperative neurophysiologic monitoring of the spinal cord. J. Clin. Neurophysiol..

[53] Murkin J.M., Arango M. (2009). Near-infrared spectroscopy as an index of brain and tissue oxygenation. Br. J. Anaesth..

[54] Highton D., Elwell C., Smith M. (2010). Noninvasive cerebral oximetry: Is there light at the end of the tunnel?. Curr. Opin. Anaesthesiol..

[55] Davie S.N., Grocott H.P. (2012). Impact of extracranial contamination on regional cerebral oxygen saturation: A comparison of three cerebral oximetry technologies. Anesthesiology.

[56] Ghosh A., Elwell C., Smith M. (2012). Review article: Cerebral near-infrared spectroscopy in adults: A work in progress. Anesth. Analg..

[57] Zhao M., Deng X., Zhang D., Wang S., Zhang Y., Wang R., Zhao J. (2019). Risk factors for and outcomes of postoperative complications in adult patients with moyamoya disease. J. Neurosurg..

[58] Fujimura M., Mugikura S., Kaneta T., Shimizu H., Tominaga T. (2009). Incidence and risk factors for symptomatic cerebral hyperperfusion following superficial temporal artery–middle cerebral artery anastomosis in patients with moyamoya disease. Surg. Neurol..

[59] Kaku Y., Iihara K., Nakajima N., Kataoka H., Fukuda K., Masuoka J., Fukushima K., Iida H., Hashimoto N. (2012). Cerebral blood flow and metabolism of hyperperfusion after cerebral revascularization in patients with moyamoya disease. J. Cereb. Blood Flow Metab..

[60] Hayashi T., Shirane R., Fujimura M., Tominaga T. (2010). Postoperative neurological deterioration in pediatric moyamoya disease: Watershed shift and hyperperfusion. J. Neurosurg. Pediatr..

[61] Uchino H., Kuroda S., Hirata K., Shiga T., Houkin K., Tamaki N. (2012). Predictors and clinical features of postoperative hyperperfusion after surgical revascularization for moyamoya disease: A serial single photon emission CT/positron emission tomography study. Stroke.

[62] Liu K., He L. (2025). Post-anesthesia care unit delirium in children with moyamoya disease undergoing indirect revascularization: Incidence and risk factors. Korean J. Anesthesiol..

[63] Chauhan V., Garcia P.S., Connolly E.S., Berman M. (2022). A retrospective review of anesthetic management of EDAS surgery for moyamoya disease with EEG and SSEP neuromonitoring. Anesth. Analg..

